# Severe Hypoglycemia Contributing to Cognitive Dysfunction in Diabetic Mice Is Associated With Pericyte and Blood–Brain Barrier Dysfunction

**DOI:** 10.3389/fnagi.2021.775244

**Published:** 2021-11-26

**Authors:** Lu Lin, Yubin Wu, Zhou Chen, Lishan Huang, Lijing Wang, Libin Liu

**Affiliations:** ^1^Department of Endocrinology, Fujian Medical University Union Hospital, Fuzhou, China; ^2^Department of Clinical Pharmacy and Pharmacy Administration, School of Pharmacy, Fujian Medical University, Fuzhou, China

**Keywords:** severe hypoglycemia, diabetes mellitus, cognitive dysfunction, pericyte, blood–brain barrier

## Abstract

**Background:** Severe hypoglycemia can cause cognitive impairment in diabetic patients, but the underlying molecular mechanism remains unclear.

**Objective:** To assess the effect of severe hypoglycemia on cognitive function in diabetic mice to clarify the relationship between the mechanism and dysfunction of pericytes and the blood–brain barrier (BBB).

**Method:** We established type 1 diabetes mellitus in 80 male C57BL/6J mice by intraperitoneal injection of streptozotocin (150 mg/kg). Further intraperitoneal injection of short-acting insulin induced severe hypoglycemia. The mice were divided into normal, diabetes, and diabetic + severe hypoglycemia groups, and their blood glucose and general weight index were examined. Pericyte and BBB morphology and function were detected by histological and western blot analyses, BBB permeability was detected by Evans blue staining, and cognitive function was detected with the Morris water maze.

**Results:** Severe hypoglycemia aggravated the histological damage, BBB damage, brain edema, and pericyte loss in the diabetic mice. It also reduced the expression of the BBB tight junction proteins occludin and claudin-5, the expression of the pericyte-specific markers PDGFR-β (platelet-derived growth factor receptor-β) and α-SMA, and increased the expression of the inflammatory factor MMP9. At the same time, diabetic mice with severe hypoglycemia had significantly reduced cognitive function.

**Conclusion:** Severe hypoglycemia leads to cognitive dysfunction in diabetic mice, and its possible mechanism is related to pericyte dysfunction and BBB destruction.

## Introduction

According to the latest data (Saeedi et al., 2020), it is estimated that by 2045, the number of diabetic patients will reach 700.2 million, and the prevalence of diabetes is rapidly rising. Hypoglycemia, hyperglycemia, and high blood glucose fluctuations are the three main characteristics in diabetes management (Monnier et al., 2012). One important therapeutic approach for treating diabetes is maintaining normal blood glucose levels and reducing the occurrence of hypoglycemia. Cognitive function refers to all aspects of thinking and intellectual activities, and is fundamental to everyday activities. Cognitive dysfunction refers to different degrees of cognitive impairment stemming from various causes, and includes mild cognitive dysfunction and dementia (Srikanth et al., 2020). Even minor changes in cognitive function potentially affect a person’s ability to achieve the best functional state, while severe cognitive dysfunction can severely reduce the patient’s quality of life and can even be life-threatening. Although epidemiological evidence shows that diabetes is closely related to cognitive dysfunction (van Sloten et al., 2020), this result remains controversial, and the exact mechanism is unknown. Many molecular and pathological consequences of diabetes overlap with factors that may lead to dementia (Biessels et al., 2020), such as changes in insulin signaling, advanced glycosylation products, and chronic low-grade inflammation, all of which are common potential mechanisms for neurological degeneration (Kullmann et al., 2020). However, an increasing number of studies have suggested that hypoglycemia plays an important role in the pathogenesis of cognitive dysfunction in diabetic patients (Lee et al., 2018; Abdelhafiz and Sinclair, 2019). Short-term mild hypoglycemia can cause reversible cognitive function impairment, while sustained or severe hypoglycemia can cause permanent neuronal damage, which further damages the brain structure and leads to changes in cognitive function (Jackson et al., 2018). However, the pathogenesis of cognitive dysfunction caused by severe hypoglycemia remains poorly understood.

Alzheimer’s disease (AD) is the most common form of dementia, and there are no therapies to slow its progression or delay its onset. Regarding AD pathogenesis, amyloid-β protein (Aβ) and tau protein immediately come to mind. With the successive failure of drugs targeting both proteins, it has become clear that the deposition of Aβ protein and tau protein may not clarify all AD pathogenesis (Sweeney et al., 2018). The Zlokovic team showed that AD patients have increased blood–brain barrier (BBB) permeability, and detected the level of soluble platelet-derived growth factor receptor-β (PDGFR-β) (a biomarker of pericyte damage) in cerebrospinal fluid (Montagne et al., 2020). It is worth mentioning that a recent study using a rat model of cerebral small vessel disease showed that restoring BBB integrity by infusing pericyte precursor cells improved cognitive function (Nakazaki et al., 2019). In summary, pericyte dysfunction, characterized by BBB destruction, may be involved in the pathological process of cognitive dysfunction-related diseases. Epidemiological and animal studies have found that diabetic patients have enhanced BBB permeability, which is related to the loss of pericyte function (Hawkins et al., 2007; Janelidze et al., 2017). However, Maarja et al. (Mae et al., 2018) found that long-term systemic hyperglycemia alone did not cause enhanced BBB permeability and impaired pericellular function in Ins2AKITA genetic diabetic mice. The authors suggested that, in addition to hyperglycemia, other features of diabetes, such as the hyperglycemia–hypoglycemia cycle, should also be considered as possible causative factors. Hypoglycemic coma significantly increased BBB permeability in the cerebral cortex, cerebellum, and brainstem regions (Yorulmaz et al., 2011). In addition, acute severe hypoglycemia-induced inflammation may destroy the BBB by reducing the expression of tight junction proteins (Zhao et al., 2018). However, the relationship and mechanism between hypoglycemia and BBB are unclear.

In recent years, the relationship between impaired BBB/pericyte function and cognitive dysfunction has attracted increased attention. As the chain of evidence from the above studies continues to be updated, it is reasonable to speculate that severe hypoglycemia plays an important role in the pathogenesis of cognitive dysfunction in diabetic patients and that this role is associated with impaired BBB/pericyte function. Nevertheless, there is no research on the issue.

In the present study, we investigated the effect of severe hypoglycemia on cognitive function in diabetic mice to clarify the relationship between decreased cognitive function and pericyte and BBB dysfunction due to severe hypoglycemia. Our findings will aid the elucidation of the relationship and related mechanisms between severe hypoglycemia and cognitive dysfunction.

## Materials and Methods

### Experimental Animal Experimental Design

Male C57BL/6J mice (*n* = 80, 20–25 g) were purchased from Zhejiang Experimental Animal Center. All experimental steps were conducted in accordance with the Animal Care and Use Guidelines of the China Experimental Society and were approved by the Fujian Animal Research Ethics Commission (Approval No. FJMU IACUC 2018-060). The animals were kept under controlled temperature and humidity in a 12-h dark–light cycle (6:00 h, lights on; 18:00 h, lights off), and food and water were freely available. After 1 week of adaptation, the mice were divided into three groups using the random number method: normal group (NC, *n* = 26), diabetic group (DM, *n* = 27), and diabetic + severe hypoglycemia group (DH, *n* = 27). The DM and DH groups were treated with a single intraperitoneal (ip) injection of streptozotocin (STZ; S0130; Sigma, St. Louis, MO, United States) dissolved in 1% (w/v) solution of 0.1 M citrate buffer (pH 4.2–4.5) at a dose of 150 mg/kg. The NC group was injected with an equal amount of citrate buffer. On day 3 after STZ injection, random blood glucose levels were measured using a FreeStyle glucometer (Abbott, Berkshire, United Kingdom) to determine whether diabetes had been successfully induced. Mice with three consecutive randomized blood glucose levels of ≥16.7 mmol/L and diabetic behavior changes (increased dietary intake, increased urination, weight loss) were considered successful in type 1 diabetes mimicry (Zhou et al., 2018a; Huang et al., 2020). Failed mice received a second STZ injection, and blood glucose testing was repeated. All mice in this study were successfully molded after the first STZ injection.

The diabetic mouse severe hypoglycemia model was established referring to the previous model of our group (Huang et al., 2020). After 8-h fast overnight, the DH group received injections of short-acting insulin (Wanbang, Jiangsu, China, dose 15 mU/g ip); the NC and DM groups received equal volumes of saline under the same conditions. Tail vein glucose was assessed every 30 min to ensure that the mice maintained low blood glucose levels (<2.0 mmol/L) over 90 min. To terminate hypoglycemia, the mice were allowed to feed or were injected with glucose (1 mg/kg ip). One mouse in the DM group and three mice in the DH group died after the modeling.

### Histologic Analyses

#### Hematoxylin–Eosin (HE) Staining

**FIGURE 1:**
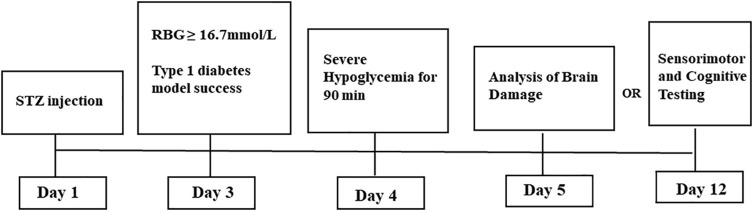
Experimental protocol.

After 24-h severe hypoglycemia modeling, 46 mice from all three groups (NC, *n* = 16; DM, *n* = 16; DH, *n* = 14)was sacrificed for histological testing; the remaining mice (NC, *n* = 10; DM, *n* = 10; DH, *n* = 10) underwent cognitive function testing in a Morris water maze test on day 8 of severe hypoglycemia (Figure 1). Morris water maze test were done after 7 days to allow the mice to recover sufficiently and to exclude factors such as lethargy and bad mood due to hypoglycemia from affecting the effect of the Morris water maze test (Jackson et al., 2018).

The mice were anesthetized with 2% sodium pentobarbital (2 ml/kg ip), and the brains were quickly removed on ice, fixed in 4% paraformaldehyde for 48 h, and embedded in paraffin. The paraffin sections were dewaxed to water, sequentially stained with hematoxylin, stained with eosin, sealed by dehydration, and examined under a microscope. The HE-stained hippocampal neurons were observed, and their images were acquired for analysis.

#### Immunohistochemistry and Immunofluorescence Staining

PDGFR-β expression levels in the mouse brain tissue were detected with immunohistochemistry. Paraformaldehyde-fixed, paraffin-embedded specimens were removed, autoclaved, heat-treated in citrate saline buffer to extract antigens, and incubated with rabbit antibody (anti-PDGFR-β, 1:200, ab32570, Abcam) overnight at 4°C. All steps were performed according to the manufacturer’s protocols. Positive staining results were brown. Images of the immunohistochemically stained sections were analyzed with Image-Pro Plus 6.0 (Media Cybernetics, Silver Spring, MD, United States).

PDGFR-β expression levels in the mouse brain tissue were examined with immunofluorescence staining. Paraffin sections were dewaxed to water, antigen-repaired, and serum-blocked. Then, the blocking solution was gently shaken off, primary antibody (anti-PDGFR-β, 1:200, ab32570, Abcam) was added dropwise to the sections, and the sections were incubated flat in a wet box overnight at 4°C. Then, the samples were incubated with secondary antibody before the nuclei were restained with DAPI. Next, the autofluorescence of the tissue was quenched, the sections were sealed, and the images were obtained under a fluorescent microscope. The DAPI-stained nuclei were blue under UV excitation and positively expressed as luciferin-labeled green fluorescence.

#### Transmission Electron Microscope Examination

Fresh mouse brain hippocampal tissue (1 mm^3^) was obtained within 1–3 min to minimize mechanical damage such as pulling, contusion, and extrusion. The brain tissue blocks were transferred to Eppendorf tubes with fresh electrodense fixing fluid and stored and transported at 4°C. The samples were rinsed three times with 0.1 M phosphate buffer (pH 7.4) for 15 min each time. Then, they were fixed, dehydrated at room temperature, and infiltration-embedded, and the embedding board was placed in a 60°C oven for 48-h polymerization. Subsequently, ultra-thin slices were obtained and stained. The sections were observed under a transmission electron microscope, and the acquired images were analyzed using Image-Pro Plus 6.0 (Media Cybernetics).

### Evaluation of Blood–Brain Barrier Permeability

A 2% concentration of Evans blue solution (E2129, Sigma) was prepared in advance with saline and injected through the mouse tail vein at a dose of 4 ml/kg at 18 h after severe hypoglycemia. After 6-h *in vivo* circulation, the mice were anesthetized and perfused with phosphate-buffered saline through the heart to remove residual Evans blue from the blood vessels. Then, the heads were removed, and the brain tissue was photographed with a Nikon D750 camera (Tokyo, Japan). Next, the brains were homogenized in 50% trichloroacetic acid solution (T5159, Sigma) and centrifuged at 20,000 × *g* for 20 min. The supernatant was collected and mixed with anhydrous ethanol (1:3). Evans blue extravasation was quantified by measuring the fluorescence intensity (excitation wavelength, 620 nm; emission wavelength, 680 nm). The amount of Evans blue dye was calculated using a standard curve and expressed as μg/g brain tissue.

### Measurement of Brain Water Content

Cortical water content is a sensitive indicator of brain edema and can be used for assessing hypoglycemia-induced brain edema and disruption of BBB function. After the mice had been anesthetized with isoflurane and sacrificed, the brains were quickly removed, and the cerebellum and olfactory bulb were discarded. The brain tissue was immediately weighed to obtain the wet weight (W), then oven-dried at 65°C for 72 h and re-weighed to obtain the dry weight (D). The brain water content (%) was calculated as (W – D)/W × 100%.

### Western Blot Analysis

After the remaining mice in each group had been euthanized, the brains were quickly removed from the ice surface, the hippocampus on both sides was separated and placed in lyophilization tubes and then quickly immersed in liquid nitrogen, the tissues were rapidly frozen and then immediately transferred to an −80°C refrigerator for storage, and set aside for subsequent testing. The protein concentration in the supernatant was detected using a bicinchoninic acid assay kit (Beyotime, Shanghai, China) according to the manufacturer’s instructions. Proteins were separated using 10% sodium dodecyl sulfate–polyacrylamide gel electrophoresis. The primary antibodies used were as follows: PDGFR-β (1:1,000, ab32570, Abcam), α-SMA (1:1,000, ab7871, Abcam), occludin (1:1,000, ab236127, Abcam), claudin-5 (1:1,000, ab131259, Abcam), MMP9 (1:1,000, GB11132, Servicebio), β-actin (1:2,000, ab8226, Abcam), and GAPDH (1:2,000, ab8245, Abcam). The membranes were then incubated with horseradish peroxidase-conjugated anti-rabbit or anti-mouse IgG (1:3,000; Cell Signaling Technology) for 1 h at room temperature and visualized by exposure to Kodak film after detection with chemiluminescent reagent (Millipore). The strip density was quantified with ImageJ analysis software.

### Grip Strength Test

To confirm that severe hypoglycemia did not impair muscle strength or flexibility, the mice underwent grip strength tests three times before the water maze test; the time spent hanging from a wire was recorded.

### Morris Water Maze

The Morris water maze consists of a 120-cm diameter, 30-cm high cylinder with a controlled water temperature of around 22°C. One week after severe hypoglycemia, the swimming trajectories of the mice (*n* = 10 per group) were analyzed by a camera tracking software system. In the hidden platform experiment, the platform with a diameter of about 7 cm was located 1.5 cm below the water surface. The mice were trained to swim for five consecutive days, four times a day. The quadrant order of each daily water entry was determined based on semi-random distribution numbers. The maximum duration of each training was 60 s. If the mice could not find the hidden platform within the specified time, the latency period was recorded as 60 s, and they were guided to stay and rest on the platform for 15 s. Swimming distance, speed, and trajectory were recorded by the system camera. The platform was removed 24 h after the last day of hidden platform training. The mice entered the water from the opposite quadrant to the original platform, and the camera tracking system recorded the time taken, the number of times the mice crossed the original platform, and the swimming path in each quadrant within 60 s.

### Statistical Analysis

All experimental data were statistically analyzed using SPSS 25.0 statistical software. Data from the hidden platform experiment were analyzed using repeated-measure two-way analysis of variance (ANOVA). The chi-square of the experimentally normally distributed measures was tested using Levene’s test (0.05). The sample means of each group that met the chi-square test requirements were compared using one-way ANOVA and LDS tests; the Kruskal-Wallis *H* test was used for data that did not meet the chi-square test requirements. Experimental data are expressed as the mean ± SEM, and *P* < 0.05 represents statistically significant differences.

## Results

### Physical and Biochemical Parameters

**FIGURE 2:**
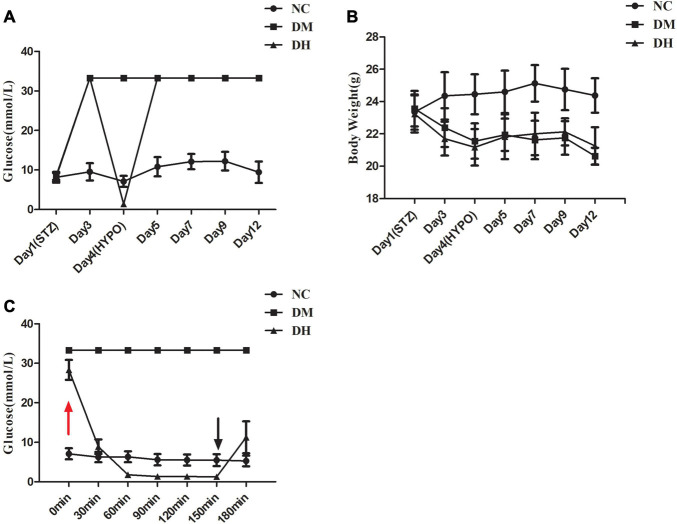
Blood glucose and body weight data. **(A)** Glycemia levels of the three groups. **(B)** Body weight trajectory of the three groups. **(C)** Glucose levels of the three groups during the severe hypoglycemia episode (<2.0 mmol/L). NC, normal control; DM, type 1 diabetes mellitus; DH, severe hypoglycemia. Data are the means ± SEM.

Compared with the NC group, mice in the DM and DH groups had significantly higher blood glucose at day 3 after STZ injection (Figure 2A). DM and DH mice had significantly decreased body weight from baseline (Figure 2B). In addition, both DM and DH mice also showed obvious symptoms of polyuria, polydipsia, and polydipsia, indicating successful establishment of the type 1 diabetes model. Figure 2C shows that the DH group started ip insulin injection at 0 min to induce the occurrence of severe hypoglycemia (red arrow), and that blood glucose dropped significantly 30 min after the injection. During severe hypoglycemia, the glucose levels in the DH mice were maintained at <2.0 mmol/L and the status lasted for 90 min (60–150 min). The hypoglycemic episode was terminated by ip injection of glucose at 150 min, and free feeding was allowed (black arrow). The next day, tail vein blood glucose was measured in each group, and blood glucose in the DH group returned to the level before severe hypoglycemia (Figure 2A).

### Severe Hypoglycemia in Diabetic Conditions Caused Histological Alterations in Hippocampal Neurons

**FIGURE 3:**
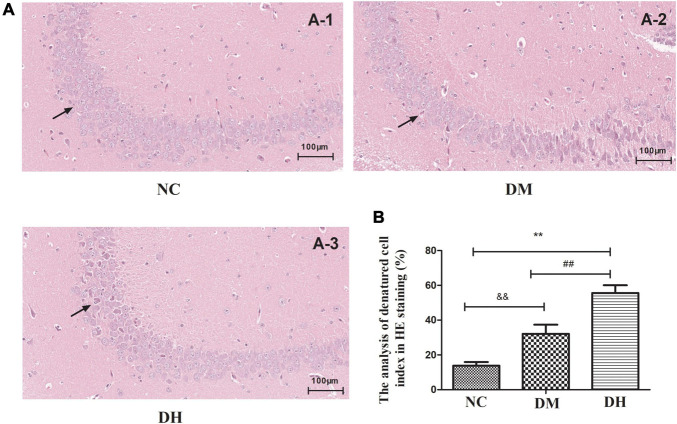
Severe hypoglycemia under diabetic conditions causes histological changes in the hippocampal region. The black arrowheads indicate layers of pyramidal cells. **(A-1)**: NC; **(A-2)**: DM; **(A-3)**: DH. Scale bar = 100 μm (HE staining). *N* = 3 per group. Data are the means ± SEM. **P < 0.01 NC vs. DH, ^&&^*P* < 0.01 NC vs. DM, ^##^*P* < 0.01 DM vs.DH.

We used HE staining to assess the histological alterations of the mouse hippocampal neurons. The NC group had regular hippocampal neuron structure, and there were 4–5 layers of pyramidal cells neatly and closely arranged, surrounded by red cytoplasm at the periphery. A large and round nucleus could be observed in the vertebral cells, and 1–2 nucleoli could be clearly seen in the nucleus (Figure 3A-1). In contrast, the DH group had disorganized hippocampal pyramidal cells, with shrunken cytosomes and shrunken or absent nuclei (NC vs. DH, *P* < 0.01; Figures 3A-3, B). The DM group had some degree of damage as compared with the NC group (NC vs. DM, *P* < 0.01; Figures 3A-2, B), but the morphological condition of the cone cells was better than that of the DH group (DM vs. DH, *P* < 0.01; Figures 3A-2, B).

### Severe Hypoglycemia Caused Blood–Brain Barrier Destruction and Pericyte Loss

#### Immunohistochemical and Immunofluorescence Staining

**FIGURE 4:**
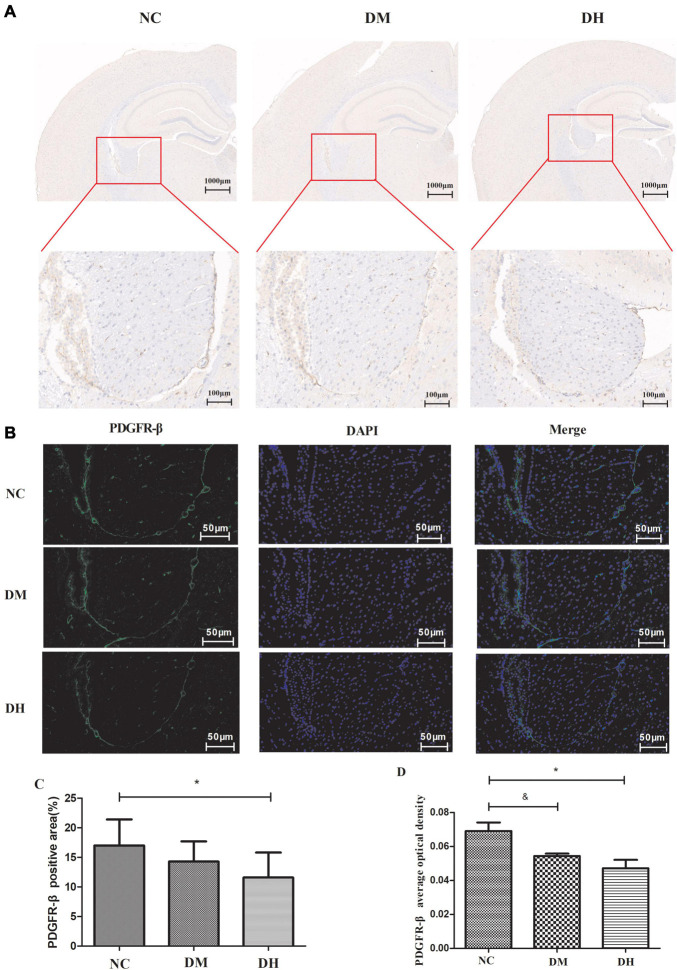
Effect of severe hypoglycemia on PDGFR-β expression in diabetic mouse hippocampus. **(A)** Representative images of immunohistochemical photographs of PDGFR-β in the brain. **(B)** Quantitation of PDGFR-β accumulation. **(C)** PDGFR-β positive expression is observed as green fluorescence; DAPI-stained nuclei are blue under UV excitation. **(D)** Comparison of quantitative analysis of mean optical density in the mice with positive PDGFR-β expression in brain capillary mid-peripheral cells in the hippocampus. Scale bar = 50 μm. *N* = 3 per group. Data are the means ± SEM. ^&^*P* < 0.05 NC vs. DM, *P < 0.05 NC vs. DH.

We used immunohistochemistry and immunofluorescence staining to observe the expression of the pericyte-specific antibody PDGFR-β in mouse brain tissue. Immunohistochemistry (Figures 4A,C) showed that the DH group had significantly reduced PDGFR-β expression compared to the NC group (*P* < 0.05), while no significant difference was seen in the DM group (*P* > 0.05). Immunofluorescence staining (Figures 4B,D) showed that PDGFR-β protein was stably expressed along the surface of intracerebral microvessels in the NC group. Compared with the NC group, the DM and DH groups had decreased PDGFR-β protein expression on the surface of the brain microvessels, and the decrease in the DH group was the most obvious. The mean optical density of PDGFR-β positivity was quantified and was significantly lower in the DM and DH groups compared to the NC group (NC, 0.069 ± 0.009 vs. DM, 0.054 ± 0.002, *P* < 0.05; NC, 0.069 ± 0.009 vs. DH, 0.047 ± 0.009, *P* < 0.05; Figure 4D).

#### Transmission Electron Microscopy

**FIGURE 5:**
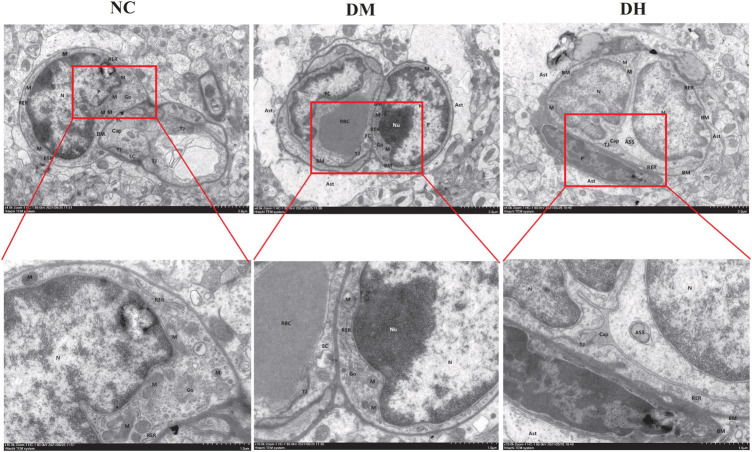
Severe hypoglycemia aggravated BBB structural damage and pericyte damage. Representative images of hippocampal BBB and pericyte morphology under transmission electron microscopy are shown. Ast, astrocyte; P, pericyte; N, nucleus; Nu, nucleolus; Go, Golgi apparatus. *N* = 3 per group.

We used transmission electron microscopy to evaluate the effects of severe hypoglycemia on BBB structure and pericyte morphology in the mice. The NC group had normal BBB, and the endothelial cells (EC) were flat and long shuttle-shaped with long nuclei, with intact nuclear membranes and normal perinuclear gaps (Figure 5). The mitochondrial membranes were intact, with normal-colored intramembrane matrix and the presence of cristae. The rough endoplasmic reticulum (RER) was not dilated, ribosome attachment was visible on the surface, and the Golgi apparatus was not proliferated or hypertrophied. Myelin-like structures were seen in the capillary lumen (Cap), and the basement membrane (BM) was continuous, intact, and of uniform thickness. There was a high number of intercellular tight junctions (TJ), and narrow intercellular space. Under further magnification, the pericytes were not edematous, the stroma was uniform, the nuclei were irregularly shaped, the nuclear membrane was intact, and the perinuclear gaps were normal. Compared with the NC group, the DH group showed severe BBB damage, severe EC edema, severe mitochondrial swelling, intramembrane matrix lysis, and cristae reduction and disappearance. The RER was markedly dilated and locally degranulated. The Cap was markedly atrophied and collapsed. The BM structure was blurred. The intercellular TJ were lost and the cell gaps were narrowed. The astrocyte footplate was markedly edematous, with sparse stroma. Further magnification revealed severe pericyte edema and intracellular low-electron density edema. The nuclei were irregularly shaped, the perinuclear gap was widened, and the heterochromatin boundary was set. Compared with the DH group, the DM group had milder impairment of the above indicators and mild BBB and pericyte impairment.

### Severe Hypoglycemia Aggravated Blood–Brain Barrier Evans Blue Leakage and Brain Edema

**FIGURE 6:**
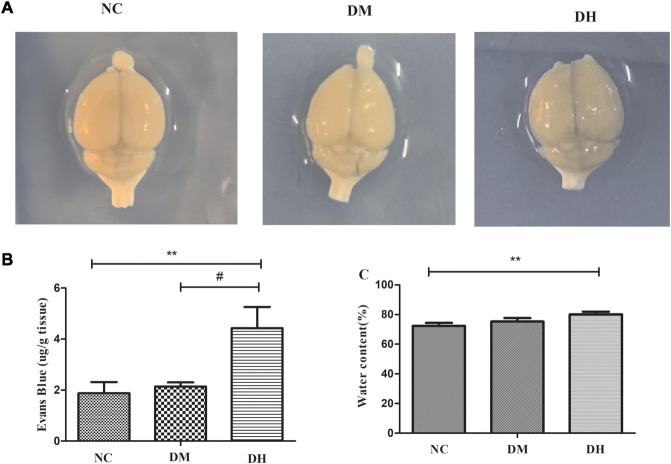
Effects of severe hypoglycemia on BBB and brain edema. **(A)** Naked-eye view of Evans blue exocytosis in mouse brain. **(B)** Quantification of Evans blue extravasation. **(C)** Mouse brain water content. *N* = 3 per group. Data are the means ± SEM. **P < 0.01 NC vs. DH, ^&^*P* < 0.05 NC vs. DM, ^#^*P* < 0.05 DM vs. DH.

We assessed the effect of severe hypoglycemia on BBB permeability in the mice using Evans blue, and quantified the Evans blue extravasation and brain water content in mice under severe hypoglycemia. The NC and DM groups brains were not stained with Evans blue, and the DH group had obvious penetration of Evans blue (Figure 6A). Quantification of Evans blue in the brain revealed that the DH group had significantly higher Evans blue content than the other two groups (NC vs. DH, *P* < 0.01, DM vs. DH, *P* < 0.05; Figure 6B), suggesting that severe hypoglycemia significantly aggravates BBB disruption in diabetic mice. The water content in the brain tissue was calculated by weighing the weight of the brain tissue before and after drying. The results showed that the DH group had significantly higher brain water content compared with the NC group (NC, DM, DH: 72.4 ± 1.9%, 75.3 ± 2.3%, 80.2 ± 1.8%, respectively; NC vs. DH, *P* < 0.01), suggesting that severe hypoglycemia aggravates brain edema in diabetic mice.

### Occludin, Claudin-5, MMP9, PDGFR-β, and α-SMA Expression After Severe Hypoglycemia

**FIGURE 7:**
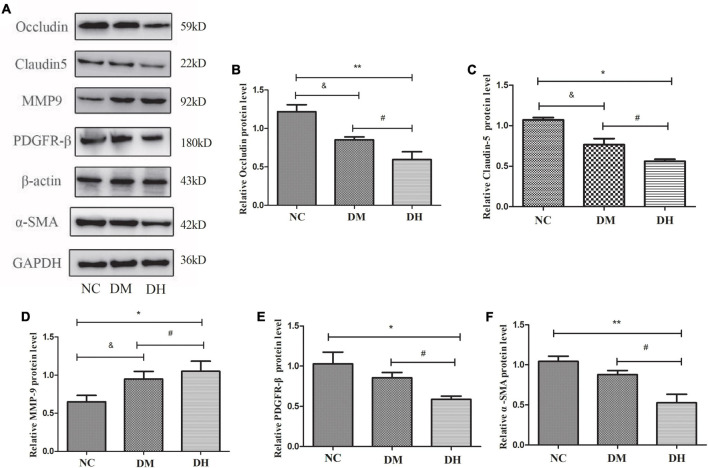
Severe hypoglycemia caused reduced expression of BBB TJ binding proteins and pericyte-specific proteins. **(A)** Representative immunoblot images of relative protein expression. Western blot analysis was performed for the protein levels of occludin **(B)**, claudin-5 **(C)**, MMP9 **(D)**, PDGFR-β (E), and α-SMA **(F)**. *N* = 3 per group. Data are the means ± SEM. ^&^*P* < 0.05 NC vs. DM. *P < 0.05 NC vs. DH, **P < 0.01 NC vs. DH, ^#^*P* < 0.05 DM vs. DH.

The expression levels of the BBB TJ proteins occludin and claudin-5, the pericyte-specific proteins PDGFR-β and α-SMA, and the pericyte injury-related protein MMP9 in the mouse hippocampus were evaluated with western blotting. Compared with the NC group, the DM and DH groups had reduced occludin and claudin-5, which were significantly decreased in the DH group (NC vs. DM, *P* < 0.05, NC vs. DH, *P* < 0.01; Figures 7A–C). The DH group also had significantly decreased occludin and claudin-5 compared with the DM group (*P* < 0.05; Figures 6A–C). Figures 7A,D show that both the DM and DH groups had increased expression of the unstable matrix metalloproteinase MMP9, associated with pericyte function (NC vs. DM, *P* < 0.05, NC vs. DH, *P* < 0.05). The DH group had significantly higher MMP9 expression compared with the DM group (*P* < 0.05; Figures 7A,D). The DH group had significantly decreased PDGFR-β and α-SMA expression compared with the NC and DM groups (PDGFR-β, NC vs. DH, *P* < 0.05, DM vs. DH, *P* < 0.05; α-SMA, NC vs. DH, *P* < 0.01, DM vs. DH, *P* < 0.05; Figures 7A,E,F).

### Severe Hypoglycemia Aggravated Cognitive Dysfunction

**FIGURE 8:**
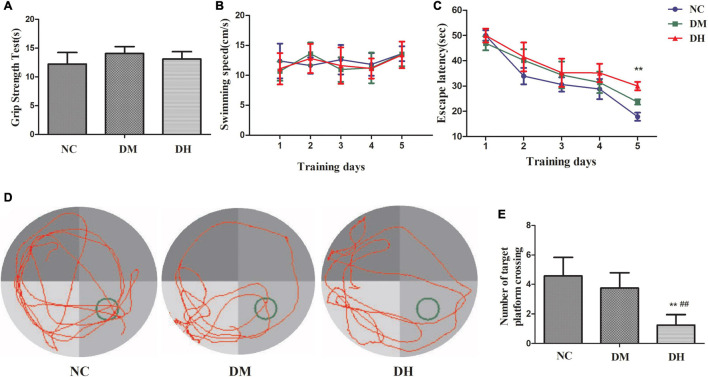
Severe hypoglycemia caused cognitive dysfunction. **(A)** Sensorimotor testing by grip strength test. **(B)** Escape latency. **(C)** Swimming speed. **(D)** Representative traces of swimming paths of each group in the probe test. **(E)** Crossings of the target quadrant during the probe trial. *N* = 10 per group. Data are the means ± SEM. **P < 0.01 NC vs. DH, ^##^*P* < 0.01 DM vs. DH.

Seven days after severe hypoglycemia, the mice was tested for sensorimotor deficits and spatial learning. Sensorimotor testing by the grip strength test showed no statistically significant difference in the duration of wire gripping between the three groups (NC, DM, DH: 12.2 ± 2.1 s, 14.1 ± 1.2 s, 13.1 ± 1.3 s, respectively, *P* > 0.05; Figure 8A). The effect of severe hypoglycemia on the learning ability of visuospatial memory in the diabetic mice was evaluated using the classical Morris water maze behavioral experiment. No significant difference was seen between the swimming speeds of the three groups (*P* > 0.05, Figure 8B). The escape latency (time spent searching for a hidden platform) gradually decreased as the training days increased (Figure 8C). The DH group had significantly longer escape latency compared to the NC group (*P* < 0.01, Figure 8C); the DM group was not significantly different compared to the NC group (*P* > 0.05). In the spatial exploration experiment with the platform removed, the DH mice passed the original platform location significantly less often compared to the NC and DM mice (*P* < 0.01, Figures 8D,E).

## Discussion

The occurrence of hypoglycemia is common in the daily glucose management of diabetic patients, and a growing number of studies have suggested that hypoglycemia plays an important role in the pathogenesis of cognitive dysfunction in diabetic patients (Bree et al., 2009; Whitmer et al., 2009; Chin et al., 2016), but the underlying molecular mechanism remains unclear. In the present study, we demonstrated that severe hypoglycemia has a significant deleterious effect on cognitive function in diabetic mice. More importantly, we demonstrate for the first time that this effect may be associated with pericyte dysfunction and BBB disruption.

Glucose is the main source of human energy and cannot be synthesized and stored in the brain, so a continuous glucose supply to the brain is essential for maintaining cognitive function. Severe hypoglycemia can cause permanent neuronal damage and further structural damage to the brain, leading to altered cognitive function (Jackson et al., 2018). Previously, we found that mitochondrial homeostasis in hippocampal tissue was imbalanced after diabetic mice experienced repeated non-severe hypoglycemia, thereby causing cognitive impairment (Zhou et al., 2018a,b). In the present study, our group continued our previous model of severe hypoglycemia in diabetic mice (Huang et al., 2020), in which a single injection of STZ caused absolute destruction of pancreatic islet β cells and clinical manifestations of type 1 diabetes mellitus with obvious “excessive drinking, polyphagia, polyuria, and weight loss.” To eliminate the influence of confounders such as physical strength and emotion of the mice after severe hypoglycemia on cognitive function testing, the mice that underwent the behavioral tests performed the grip test after a full 1-week rest. The results showed similar abilities in grip strength and swimming speed among the groups, with no measurable differences in sensorimotor coordination and strength between the groups, suggesting that the absence of gross motor impairment may affect the cognitive function of the mice. The Morris water maze subsequently demonstrated that severe hypoglycemia in the diabetic state can cause significant deficits in memory consolidation and working memory capacity in mice.

The BBB is a highly selective, semi-permeable border that separates circulating blood from extracellular fluid in the brain and central nervous system, and is formed by EC in the capillary wall, astrocyte ends wrapped around the capillaries, and pericytes embedded in the BM of the capillaries. Pericytes, a BBB component, share a BM with EC, and in regions lacking a BM, the intersection of pericytes and EC membranes, termed a peg–rivet contact, forms a direct connection that controls molecular exchange between pericytes and EC (Hort et al., 2019). Under physiological conditions, pericytes play an important role in BBB formation and maintenance, neurovascular system regulation, inflammatory cell transport, and toxic metabolite removal from the brain (Uemura et al., 2020). In recent years, the relationship between BBB function and AD has become a topic of interest. A study (van de Haar et al., 2016) showed that AD patients had greater BBB leakage compared to the healthy population, and the investigator concluded that BBB disruption is an early biomarker of cognitive dysfunction in humans. It has also been argued (Blanchard et al., 2020) that BBB integrity is lost prior to cognitive decline. Pericyte deficiency has been observed in animal models of AD and in postmortem histological studies (Nikolakopoulou et al., 2017). In another study (Nortley et al., 2019), capillaries in the brain tissue of AD patients and in mice bred to develop AD pathology were extruded by the pericytes and showed significant dysfunction. The authors calculated that this capillary constriction was sufficiently severe to halve blood flow, which is comparable to the reduction in blood flow to the part of the brain affected by AD, and they proposed that restoring perivascular cell function in the brain holds promise for treating AD. In conclusion, pericytes may play an important role in the pathogenesis of cognitive dysfunction. Therefore, is hypoglycemia-induced cognitive dysfunction associated with pericyte/BBB disruption? Studies have found that (Uemura et al., 2020) hypoglycemia potentially damages BBB integrity and function, and hypoglycemia exposure severely affected the expression of BBB oxidation and inflammatory stress markers. However, there are no studies on BBB disruption in diabetic mice after severe hypoglycemia. In the present study, diabetic mice with severe hypoglycemia showed increased Evans blue infiltration and significant brain edema, suggesting severe BBB disruption. BBB/neurovascular unit impairment is now considered one of the key factors contributing to the development of diabetic encephalopathy (Bogush et al., 2017), and diabetic mice exhibit enhanced BBB permeability after severe hypoglycemia, which may be associated with the loss of perivascular cells in the brain. To clarify the mechanism of BBB disruption, we examined the indicators related to pericyte function and distribution, and show that pericyte-specific expression of the proteins PDGFR-β and α-SMA was reduced in the brains of diabetic mice after severe hypoglycemia, suggesting that the pericytes were lost. The transmission electron microscopic findings also suggest that diabetic mice have BBB disruption and pericyte damage after severe hypoglycemia. The preliminary results of the present study suggest that the mechanism of cognitive dysfunction after severe hypoglycemia in diabetic mice may be related to BBB disruption, which may result from pericyte dysfunction and loss. Therefore, how might pericytes in hypoglycemia affect BBB function?

The presence of oxidative stress during hypoglycemia has been well demonstrated, with increased reactive oxygen species (ROS) production in mitochondria isolated from the hippocampus of diabetic rats exposed to recurrent hypoglycemia (Shukla et al., 2019). In addition, elevated levels of inflammatory factors such as TNF-α and IL-6 have been observed in hypoglycemia, causing an acute inflammatory state in the organism (Solis-Herrera et al., 2020). MMPs are zinc-dependent proteases that degrade many structural components of the extracellular matrix and non-extracellular matrix proteins (Hawkins et al., 2007), and include MMP9. Oxidative stress and an inflammatory environment contribute to MMP9 activation and secretion by pericytes, which decreases the expression of TJ proteins and leads to BBB destruction (Persidsky et al., 2016). MMP9 inhibitors reduce pericyte-associated BBB leakage (Underly et al., 2017). In the present study, MMP9 expression was increased in diabetic mice and severely hypoglycemic mice, and the levels of occludin and claudin-5, closely related to BBB function, were further decreased; ultramicroscopy of mouse brain hippocampal tissue also showed significant TJ disruption. It is suggested that severe hypoglycemia in the high-glucose state can cause increased MMP9 expression in brain pericytes, which further causes degradation of the TJ proteins and leads to BBB disruption.

Notably, in the present study, although MMP9 levels were increased and TJ proteins were decreased in the diabetic state, no further BBB disruption or reduced pericyte numbers by hyperglycemia was observed, and the water maze behavior also suggested no significant cognitive dysfunction in the diabetic mice alone. Although diabetes is associated with increased risk of neurodegeneration and dementia (Biessels et al., 2006), the specific effects of hyperglycemia on pericyte/BBB function remain controversial. Several studies have suggested (Liu et al., 2021) hat the mechanism of pericyte dysfunction and consequent BBB leakage in diabetic patients is related to enhanced ROS and reduced ATP production due to oxidative stress caused by prolonged hyperglycemia. In contrast, others have found that BBB function remained unchanged in diabetes (Mae et al., 2018). Yet, our results show that hyperglycemia alone caused a slight impairment in pericyte function and in BBB morphology and function compared to that in normal mice. However, it was insufficient to cause significant BBB disruption and subsequent cognitive dysfunction as compared to the severe hypoglycemia group. We conjecture that the possible reason for the high glucose in this model is the short duration of the study (only 5 days from sampling and 12 days from the water maze test), and the glycosylation products and oxidative stress of the high glucose were insufficient to cause serious BBB disruption and thereby lead to cognitive dysfunction. However, according to the trend of our results, it is reasonable to think that long-term hyperglycemia will destroy pericyte/BBB function. To explore the effect of hyperglycemia on pericyte/BBB function, further investigations can extend the duration of hyperglycemia in diabetic mice or use diabetic genotype mice.

It is well known that neuronal damage is a key factor in cognitive dysfunction (Tobin et al., 2019). The loss of pericytes may disrupt the BBB through the leakage and deposition of vascular and neurotoxic macromolecules from peripheral circulation sources. The associated microvascular degeneration and edema may cause chronic hypoxia by further increasing the entry of neurotoxic substances and causing reduced blood flow, resulting in structural and functional changes in neurons, thereby affecting interneuronal interconnections and ultimately exacerbating the development of primary neurodegenerative disease (Nikolakopoulou et al., 2019). Pericyte-deficient mice have increased neuronal apoptosis, reduced neuronal numbers, and behavioral deficits in the cortex and hippocampus (Kisler et al., 2017). In the present study, HE staining revealed significant neuronal destruction in the severely hypoglycemic mice, and we speculate that the cause may be the leakage and deposition of vascular and neurotoxic macromolecules after pericyte/BBB dysfunction that further exacerbate neuronal damage, thereby causing cognitive dysfunction. The specific molecular mechanism requires further research.

This study has some limitations. First, this study used STZ injection to create a diabetic mice model, and it was reported that STZ injection could directly cause brain damage (Liu et al., 2020).

However, in the present study, mice in the group that experienced severe hypoglycemia had significantly more impairment in cognitive function and BBB compared with mice in the diabetic group that received STZ injection alone, suggesting an independent or additive effect of hypoglycemia on these impairments. Second, the effect of severe hypoglycemia on pericytes/the BBB has not been further explored at the cellular level, and the specific mechanisms have not been explored in depth. This is the next step of our research plan. Our study shows, for the first time, that the severe hypoglycemia-induced cognitive dysfunction in diabetic mice is associated with pericyte/BBB dysfunction.

## Conclusion

Our results suggest that severe hypoglycemia in diabetic mice can lead to the development of cognitive dysfunction. The possible mechanism is that severe hypoglycemia occurs under diabetes, which leads to pericyte dysfunction and subsequently BBB destruction. Our experimental results will aid the elucidation of the role of severe hypoglycemia and cognitive dysfunction, and also contribute to providing new biomarkers for early prediction of the development of cognitive impairment due to severe hypoglycemia.

## Data Availability Statement

The original contributions presented in the study are included in the article/supplementary material, further inquiries can be directed to the corresponding author.

## Ethics Statement

The animal study was reviewed and approved by the Fujian Animal Research Ethics Commission (Approval No. FJMU IACUC 2018-060).

## Author Contributions

LUL contributed to the conception of the study and manuscript. YBW performed the experiments and analyzed the data. LSH and LJW contributed to performing the experiments. ZC and LBL contributed to the conception of the study and manuscript preparation. All authors contributed to the article and approved the submitted version.

## Conflict of Interest

The authors declare that the research was conducted in the absence of any commercial or financial relationships that could be construed as a potential conflict of interest.

## Publisher’s Note

All claims expressed in this article are solely those of the authors and do not necessarily represent those of their affiliated organizations, or those of the publisher, the editors and the reviewers. Any product that may be evaluated in this article, or claim that may be made by its manufacturer, is not guaranteed or endorsed by the publisher.

## Abbreviations

BBB: blood–brain barrier
PDGFR-β: platelet-derived growth factor receptor-β
AD: Alzheimer’s disease
Aβ: amyloid-β protein
NC: normal group
DM: diabetic group
DH: diabetic + severe hypoglycemia group
STZ: streptozotocin
HE: hematoxylin – eosin staining
EC: endothelial cells
RER: rough endoplasmic reticulum
Cap: capillary lumen
BM: basement membrane
TJ: tight junctions
ROS: reactive oxygen species.
